# The Prevalence of Anatomical Variations of the Median Nerve in the Carpal Tunnel: A Systematic Review and Meta-Analysis

**DOI:** 10.1371/journal.pone.0136477

**Published:** 2015-08-25

**Authors:** Brandon Michael Henry, Helena Zwinczewska, Joyeeta Roy, Jens Vikse, Piravin Kumar Ramakrishnan, Jerzy A. Walocha, Krzysztof A. Tomaszewski

**Affiliations:** Department of Anatomy, Jagiellonian University Medical College, 12 Kopernika Street, 31–034, Krakow, Poland; University of Palermo, ITALY

## Abstract

**Background and Objective:**

The course and branches of the median nerve (MN) in the wrist vary widely among the population. Due to significant differences in the reported prevalence of such variations, extensive knowledge on the anatomy of the MN is essential to avoid iatrogenic nerve injury. Our aim was to determine the prevalence rates of anatomical variations of the MN in the carpal tunnel and the most common course patterns and variations in its thenar motor branch (TMB).

**Study Design:**

A systematic search of all major databases was performed to identify articles that studied the prevalence of MN variations in the carpal tunnel and the TMB. No date or language restrictions were set. Extracted data was classified according to Lanz's classification system: variations in the course of the single TMB—extraligamentous, subligamentous, and transligamentous (type 1); accessory branches of the MN at the distal carpal tunnel (type 2); high division of the MN (type 3); and the MN and its accessory branches proximal to the carpal tunnel (type 4). Pooled prevalence rates were calculated using MetaXL 2.0.

**Results:**

Thirty-one studies (n = 3918 hands) were included in the meta-analysis. The pooled prevalence rates of the extraligamentous, subligamentous, and transligamentous courses were 75.2% (95%CI:55.4%-84.7%), 13.5% (95%CI:3.6%-25.7%), and 11.3% (95%CI:2.4%-23.0%), respectively. The prevalence of Lanz group 2, 3, and 4 were 4.6% (95%CI:1.6%-9.1%), 2.6% (95%CI:0.1%-2.8%), and 2.3% (95%CI:0.3%-5.6%), respectively. Ulnar side of branching of the TMB was found in 2.1% (95%CI:0.9%-3.6%) of hands. The prevalence of hypertrophic thenar muscles over the transverse carpal ligament was 18.2% (95%CI:6.8%-33.0%). A transligamentous course of the TMB was more commonly found in hands with hypertrophic thenar muscles (23.4%, 95%CI:5.0%-43.4%) compared to those without hypertrophic musculature (1.7%, 95%CI:0%-100%). In four studies (n = 423 hands), identical bilateral course of the TMB was found in 72.3% (95%CI:58.4%-84.4%) of patients.

**Conclusions:**

Anatomical variations in the course of the TMB and the MN in the carpal tunnel are common in the population. Thus, we recommend an ulnar side approach to carpal tunnel release, with a careful layer by layer dissection, to avoid iatrogenic damage to the TMB.

## Introduction

During carpal tunnel release (CTR) surgery or repair of traumatic injuries to the wrist, careful attention must be paid to the course of the median nerve [1, 2]. Numerous variations in the course of the median nerve in the carpal tunnel, as well as variations in its branches in the wrist and in the hand, have been reported [1, 2, 3]. As such, a reliable knowledge of the anatomy of the median nerve in the wrist is essential to prevent iatrogenic damage during surgical procedures.

**Fig 1:**
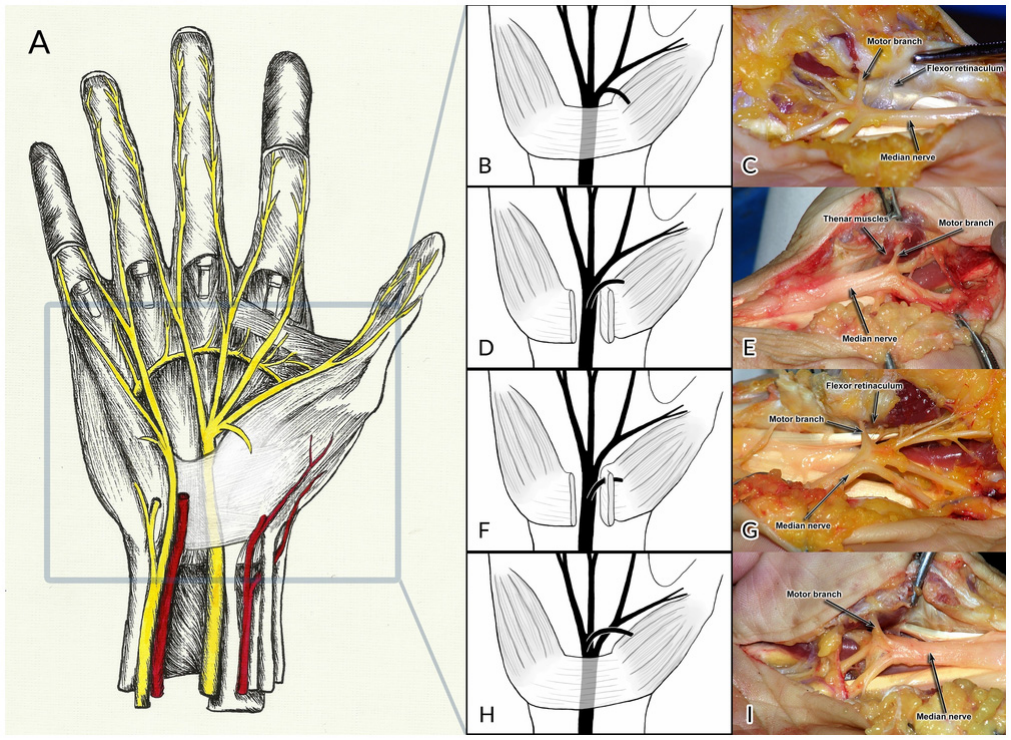
Overview of the course of the median nerve in the wrist and hand (A), extraligamentous type TMB with radial side of branching (B, C), subligamentous type TMB (D, E), transligamentous type TMB (F, G), extraligamentous type TMB with ulnar side of branching (H, I).

The median nerve, consisting of both motor and sensory fibers, originates from the spinal roots of the brachial plexus at the level of C5—T1 [4]. At the level of the wrist, the median nerve passes through the carpal tunnel, deep to the transverse carpal ligament (TCL) (Fig 1A), together with the four tendons of the flexor digitorum superficialis, the four tendons of the flexor digitorum profundus and the tendon of the flexor pollicis longus [4].

After the median nerve exits the carpal tunnel, it divides into medial and lateral branches. The medial branch terminates as two common palmar digital nerves supplying motor innervation to the second lumbrical and sensory innervation to the palm and fingers [4]. The lateral branch gives off the thenar motor branch (TMB), before giving rise to proper palmar digital nerves supplying motor innervation to the first lumbrical, and sensory innervation to the lateral side of the hand [4].

The TMB is also known as the recurrent branch of the median nerve or the "million dollar nerve" [5], due to high legal costs incurred by surgeons upon accidental damage to the nerve during surgical procedures [2]. The TMB supplies motor innervation to the opponens pollicis, the abductor pollicis brevis, and the superficial part of flexor pollicis brevis [4]. There are several frequent variations to the course of the TMB [3]. Therefore, special clinical attention should be paid by surgeons to variations of the TMB during median nerve decompression for carpal tunnel syndrome (CTS) [3]. A detailed knowledge of the particular variations of the TMB, as well as their prevalence rates, may reduce unnecessary surgical complications and prevent litigious consequences for surgeons.

Many of the previous studies on variations in the course of the TMB have used the Poisel classification system [6]. Poisel distinguished three types of TMB branching: the extraligamentous (type I), the subligamentous (type II), and the transligamentous (type III) [3]. In the extraligamentous type, which Poisel reported to be the most common, the TMB arises distal to the TCL and then takes a retrograde course to reach the thenar muscles (Fig 1B and 1C) [3, 6]. In the subligamentous type, the branch arises within the carpal tunnel and remains deep to the TCL until it reaches the distal end of the ligament and bends around it to reach the thenar muscles (Fig 1D and 1E) [3, 6]. In the third variation, the transligamentous type, the branch arises within the carpal tunnel and pierces the TCL to reach the thenar muscles (Fig 1F and 1G) [3, 6]. As such, this variation of the TMB is at a particularly high risk for damage during CTR procedures [1].

Additionally, variations in the side of branching of the TMB have been reported [7, 8, 9]. Normally, the TMB arises from the radial or palmar side of the median nerve. However, a TMB arising from the ulnar side of the median nerve has also been reported, and is at a higher risk for inadvertent damage during CTR procedures (Fig 1H and 1I) [7, 8, 9].

Lanz expanded Poisel's original classification system, to include variations of the median nerve in the carpal tunnel [1]. He described four groups of TMB variations: variations in the course of the single TMB according to Poisel (type 1), accessory branches of the median nerve at the distal carpal tunnel (type 2), high division of the median nerve (type 3), which Lanz reported to be associated with the presence of a persistent median artery (PMA) running with the bifid median nerve, and accessory branches of the median nerve proximal to the carpal tunnel (type 4) [1].

The reported prevalence rates of Lanz group variations in the literature have varied from study to study, and the reported prevalence rates of TMB variants have varied significantly from Poisel's original description [10]. Interestingly while originally classified by Poisel, Lanz's drawings have been primarily used in most studies to describe variations in TMB. However, Lanz's drawings do not correlate exactly to Poisel's [10]. Additionally, while the use of the terms extraligamentous, subligamentous, and transligamentous have been used in studies since 1974 when first described by Poisel, different studies have used slightly different interpretations when classifying their results, making comparisons between the reported prevalence rates slightly difficult [10].

**Fig 2:**
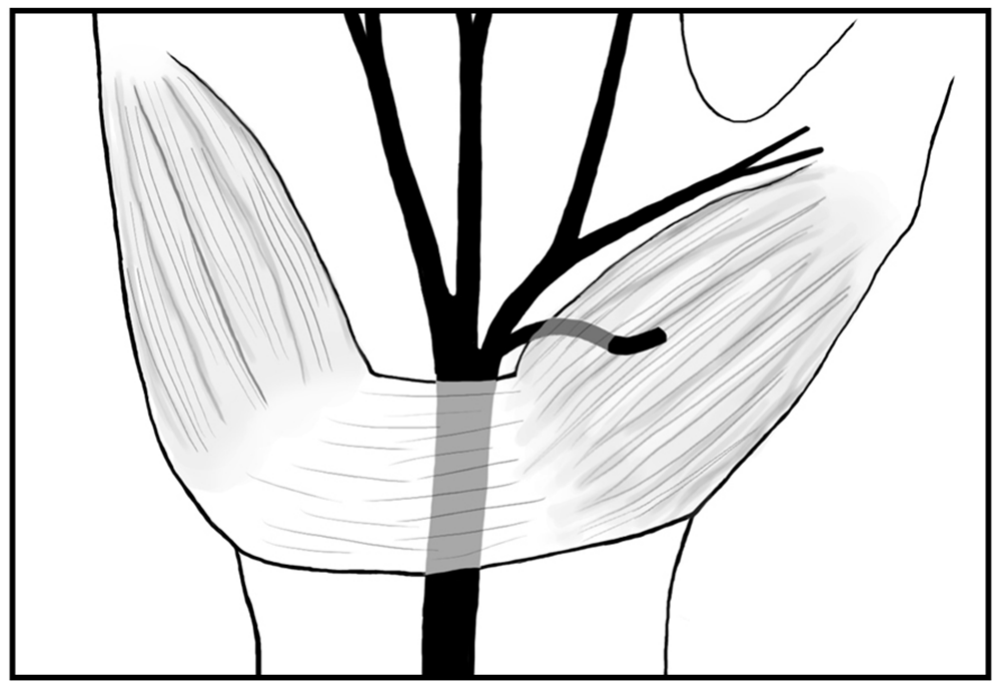
Hypertrophic thenar muscles overlying the TCL with an extraligametous type TMB running within the tissue.

Additionally, there has been significant interest in reported hypertrophic thenar musculature superficial to, or interposed within, the TCL (Fig 2) [6, 10, 11]. The exact origin of this muscle tissue is unknown, however Mannerfelt and Hybbinette reported in 1972 that the presence of such hypertrophic muscle tissue in strongly suggestive of variations in the TMB, and as such, surgeons should take extra care to locate the position of the TMB when encountering hypertrophic muscles over the TCL [6, 10].

The aim of the present study was to perform a systematic review of the literature and a meta-analysis to determine the most common course patterns and variations of the TMB and the median nerve in the carpal tunnel according to Lanz's classification. To the best knowledge of the authors, no such meta-analysis has ever been performed.

## Methods

### Search Strategy

We performed a literature search through March 2015 of the electronic databases PubMed, EMBASE, Science Direct, Scopus, Web of Science, and Cochrane Library, to identify studies eligible for the meta-analysis. To achieve a high sensitivity of search results and identify all studies, the search strategy was individually adjusted for each electronic databases. The search terms included median nerve, nervus medianus, motor branch, recurrent branch, recurrent motor branch, thenar motor branch, thenar branch, carpal tunnel, carpal canal, transverse carpal ligament, flexor retinaculum, anterior annular ligament, anatomy, variations, extraligamentous, subligamentous, transligamentous, supraligamentous, accessory branches, high division, and high bifurcation. References of articles included in the meta-analysis were searched to identify additional articles eligible for inclusion in the meta-analysis. No date or language restrictions were set. PRISMA guidelines were strictly followed throughout the search process and the meta-analysis.

### Criteria for Study Selection

Studies were considered eligible for inclusion in the meta-analysis if they (1) reported extractable prevalence data related to Lanz's classification type 1, 2, 3, or 4 or data on side of branching of the TMB, (2) had clearly defined descriptions of TMB variations, and (3) was a cadaveric or a prospective intraoperative study. Exclusion criteria included (1) missing data or incomplete data sets, (2) data not able to be classified according to the Lanz classification, and (3) retrospective intraoperative study. The decision to exclude retrospective intraoperative chart review studies was due to the lack of reliable anatomical data in such study designs, and was made only after consulting the authors of these studies. Case reports, conference abstracts, and letters to the editors, were reviewed, but not included in the meta-analysis. Each study was independently assessed by three authors (B.M.H., J.R., and J.V.) for eligibility in the meta-analysis. Any disagreements during the eligibility processes were settled by a consensus among the authors. Full-text articles in languages not spoken by the authors were translated by medical professionals fluent in both English and the original language of the manuscript, for further eligibility assessment.

### Data Extraction

Data was independently extracted by three authors (B.M.H., J.R., and J.V.) from the included studies. Data extracted included study design, country, sample size, number of hands, course of the TMB (Lanz group 1), distal and proximal accessory branches of the median nerve (Lanz groups 2 and 4), high division of the median nerve (Lanz group 3), side of branching, and symmetry of the course of the TMB between hands bilaterally. As anatomical definitions of variations differed amongst the studies, each author carefully examined the anatomical definitions of variations in each study to determine the appropriate classification of the individual study data within the Lanz classification system used in this meta-analysis. In the event of any disagreement among the authors, consultation with all the authors was made and solved by a consensus. Data on rare TMB anomalies, such as preligamentous and supraligamentous courses of TMB, was recorded from the studies but not included in the meta-analysis. In the event that a study reported a rare TMB anomaly, that hand was subtracted from the total number of hands in the study for the purposes of statistical analysis. Data on side of branching of the TMB was classified into two groups for the analysis, (1) radial/anteroradial side and (2) ulnar side. Authors were contacted by email in the event of discrepancies in the data, and further data was sought after from the authors when necessary.

### Statistical Analysis

Statistical analysis was performed by B.M.H. and P.K.R. using MetaXL version 2.0 by EpiGear International Pty Ltd (Wilston, Queensland, Australia). The frequency of anatomical variations from the individual studies were pooled into the meta-analysis using a double arcsine transformation, with a back-transformation to report the pooled prevalence rates [12]. Because of the need to calculate multi-categorical pooled prevalence rates, we chose such a transformation to stabilize the variance in our analysis. The variance in a double arcsine transformation is dependent only on the population size, and has been shown to be preferential to logit transformations in multi-categorical prevalence meta-analysis [12]. All analyzes were performed using a random effects model. The Chi^2^ test and I^2^ statistic were used to assess heterogeneity among the studies. For the Chi^2^ test, a p-value of < 0.10 was considered to indicate statistically significant heterogeneity between studies [13]. The value of the I^2^ statistic was interpreted as follows: 0% to 40% might not be important; 30% to 60% may represent moderate heterogeneity; 50% to 90% may represent substantial heterogeneity; and 75% to 100% may represent considerable heterogeneity [13]. When appropriate, subgroup analysis based on study design or geographical distribution was performed to explore the sources of heterogeneity. Statistically significant differences between 2 or more subgroups was determined through the use of confidence intervals. If the confidence intervals between two subgroups overlapped, the differences were considered insignificant. Additionally, a sensitivity analysis was also performed when appropriate to help examine heterogeneity, by limiting inclusion to studies with ≥ 100 hands.

## Results

### Study Identification

**Fig 3:**
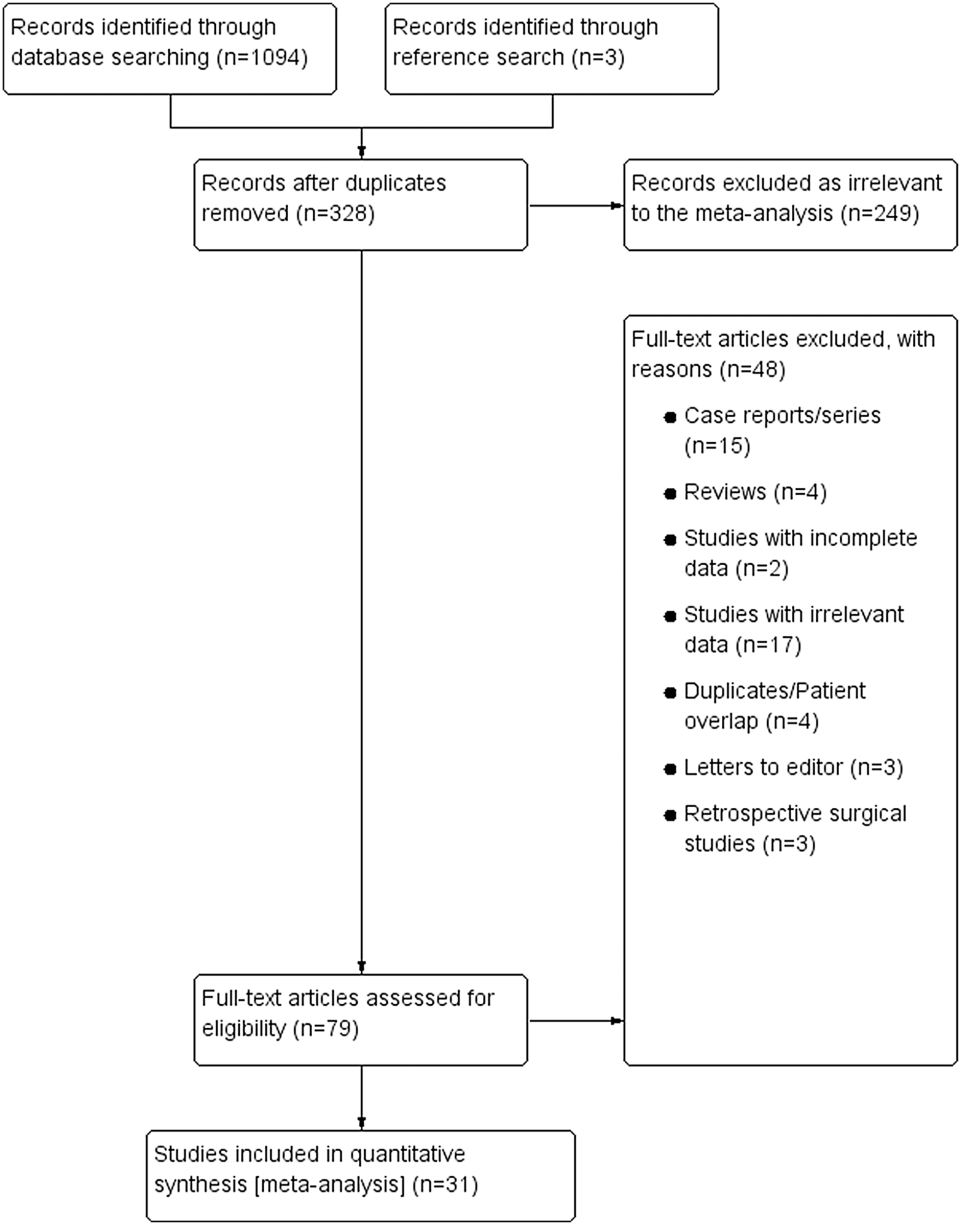
Flow chart of study identification, evaluation and inclusion in the meta-analysis.

An overview of the study identification process is summarized in Fig 3. Through database searching, 1094 articles were initially identified. A further 3 articles were identified through reference searching. After screening and removal of duplicates, 80 articles were assessed by full text for eligibility in the meta-analysis. Of the full-text articles, 31 were deemed eligible for inclusion and 49 were excluded from the meta-analysis. Two studies by Stancic et al. [14] and Vashishtha et al. [15] were excluded due to incomplete data and a lack of response from the authors for additional data.

### Characteristics of Included Studies

The included study characteristics are summarized in Table 1, which contains the prevalence data on TMB variations as reported and described in each individual study. A total of 31 studies (n = 3918 hands) were included in the meta-analysis, with 6 intraoperative studies [1, 6, 7, 10, 20, 22], 23 cadaveric studies [2, 3, 8, 9, 16–19, 21, 23–36] and 1 study [37] which included both a cadaveric and an intraoperative study group, which for the purposes of this analysis was analyzed as two individual studies. The study with both cadaveric and intraoperative groups by Tountas et al. [37], contained two separate intraoperative groups—a prospective group and a retrospective group, of which only the prospective data was included in the meta-analysis.

**Table 1:** Characteristics of the included studies in the meta-analysis.

Study	Population (Continent)	Type	n =	Lanz Group 1 (%)	Side of Branching (%)	Lanz Group II (%)	Lanz Group III (%)	Lanz Group IV (%)
**Agarwal 2014 [2]**	Indian (Asia)	C	52	Extraligamentous = 36.53 Subligamentous = 21.15 Transligamentous = 42.30		3.84	11.53	1.92
**Raviprasanna2014 [31]**	Indian (Asia)	C	51	Extraligamentous = 78.39 Subligamentous = 19.6 Preligamentous = 1.96				
**Samarakoon 2014 [33]**	Sri-Lankan (Asia)	C	26	Extraligamentous = 84.6 Subligamentous = 3.8 Transligamentous = 11.5				
**Planitzer2013 [30]**	German (Europe)	C	21		Radial dorsal side = 35 Radial palmar and dorsal side = 29 Radial palmar side = 18 Ulnar dorsal side = 12 Radial and ulnar palmar side = 6			
**Mizia 2012 [8]**	Polish (Europe)	C	60	Extraligamentous = 78.3 Subligamentous = 20 Transligamentous = 1.7	Radial side = 75 Anterior side = 10 Ulnar side = 3.33 Between ulnar and radial side = 11.67			
**Pereira 2012 [29]**	Brazilian (South America)	C	20	Extraligamentous = 90 Subligamentous = 10 Transligamentous = 0				
**Al-Qattan 2010 [6]**	Saudi Arabian (Asia)	IO	100	Extraligamentous = 56 Subligamentous = 34 Transligamentous = 9 Preligamentous = 1				
**Ozcanli 2010 [28]**	Turkish (Europe)	C	30	Extraligamentous = 60 Subligamentous = 34 Transligamentous = 6				
**Senanayake 2009 [34]**	Sri-Lankan (Asia)	C	60	Extraligamentous = 88 Subligamentous = 12 Transligamentous = 0				
**Green 2008 [10]**	American (North America)	IO	1400	Retrograde (extraligamentous) = 15 Retrograde with fenestrations = 7 Normal (subligamentous) = 72 Intramuscular (transligamentous) = 4 Muscular TCL with normal motor branch = 2				
**Alizadeh 2006 [16]**	Iranian (Asia)	C	60	Extraligamentous = 46.7 Subligamentous = 28.3 Transligamentous = 11.7 Transfacial = 13.3	Ulnar side = 12	18.4		18.4
**Caetano 2006 [19]**	Brazilian (South America)	C	30	Extraligamentous = 83.3 Subligamentous = 13.3 Transligamentous = 3.4	Ulnar side = 5 Anterior side = 20 Radial side = 75			
**Sacks 2006 [32]**	American (North America)	C	48	Extraligamentous = 92 Subligamentous = 0 Transligamentous = 8	Radial and volar side = 100 Ulnar side = 0			
**Alp 2005 [17]**	Turkish (Europe)	C	144	Proximal to TCL = 2 Distal to TCL = 84 Between borders of TCL = 14				
**Barbe 2005 [18]**	American (North America)	C	89			0	2.6	0
**Eskandari 2005 [20]**	Turkish (Europe)	IO	37	Extraligamentous = 48.65 Subligamentous = 21.6 Transligamentous = 29.73	Anteroradial = 100 Ulnar side = 0			
**Ahn 2000 [7]**	Korean (Asia)	IO	354	Extraligamentous = 96.1 Subligamentous = 2.8 Transligamentous = 1.1	Radial border = 62.2 Radial one third = 18.9 Anterior side = 17.2 Ulnar side = 1.1		0.3	
**Kozin 1998 [24]**	American (North America)	C	101	Extraligamentous = 93 Subligamentous = 0 Transligamentous = 7	Central volar position = 41 Radial to central position = 58 Direct radial position = 1 Ulnar side = 0			
**Steinberg 1998 [36]**	Israeli (Asia)	C	46	Extraligamentous = 71.74 Transligamentous = 28.26				
**Hurwitz 1996 [22]**	Swiss (Europe)	IO	80	Extraligamentous = 55 Subligamentous = 29 Transligamentous = 16	Anterior side = 12.5 Ulnar side = 1.25 Radial side = 78.75 Second division = 6.25 Dorsal side of first division = 1.25			
**Olave 1996 [27]**	Brazilian (South America)	C	60	Extraligamentous = 80 Subligamentous = 18.3 Transligamentous = 0			1.66	
**Siverhus 1989 [35]**	American (North America)	C	72	Extraligamentous = 86 Transligamentous = 14				
**Mackinnon 1988 [25]**	Canadian (North America)	C	50		Extreme radial side = 60 Extreme radial volar and central aspect = 18 Central volar position = 22 Ulnar side = 0		2	
**Tountas 1987 [37]**	American (North America)	IO	286	Extraligamentous = 95.10 Subligamentous = 2.79 Transligamentous = 1.40	Radial side = 92.3 Anterior side = 5.95 Ulnar side = 1.75	0.70	2.10	2.10
		C	92	Extraligamentous = 81.52 Subligamentous = 9.78 Transligamentous = 8.70	Radial side = 100 Anterior Side = 0 Ulnar side = 0	2.17	1.09	0
**Mumford 1986 [26]**	American (North America)	C	20	Extraligamentous = 80 Transligamentous = 20	Anteroradial side = 20 Anteroulnar side = 80			
**Falconer 1985 [21]**	American (North America)	C	10	Extraligamentous = 30 Subligamentous = 10 Transligamentous = 60		20		0
**Perneczky 1980 [9]**	French (Europe)	C	163	Extraligamentous = 51 Subligamentous = 23 Transligamentous = 26	Distal branching = 5 Ulnar side = 2			
**Lanz 1977 [1]**	German (Europe)	IO	246			7.32	2.85	1.63
**Poisel1974 [3]**	German (Europe)	C	100	Extraligamentous = 46 Subligamentous = 31 Transligamentous = 23				
**Johnson 1970 [23]**	American (North America)	C	10	Extraligamentous = 20 Transligamentous = 80				

C- cadaveric, IO—intraoperative.

### Lanz Group 1—Course of the TMB

A total of 27 studies (n = 3506 hands), 21 cadaveric [2, 3, 8, 9, 16, 17, 19, 21, 23, 24, 26–29, 31–37] and 6 intraoperative [6, 7, 10, 20, 22, 37], reported prevalence data on the course of the TMB. The most common course of the TMB was extraligamentous, with a pooled prevalence of 75.2% (95% CI: 55.4%- 84.7%). The second most common course was subligamentous with a pooled prevalence of 13.5% (95% CI: 3.6%- 25.7%), while the least common course was transligamentous with a pooled prevalence = 11.3% (95% CI: 2.4%- 23.0%). The difference between the subligamentous and transligamentous courses was not statistically significant. High heterogeneity existed among the studies (Cochran's Q, p = 0.000; I^2^ = 98.7%, 95% CI: 98.5%- 98.9%), and as such, subgroup analysis was performed to explore the cause.

**Fig 4:**
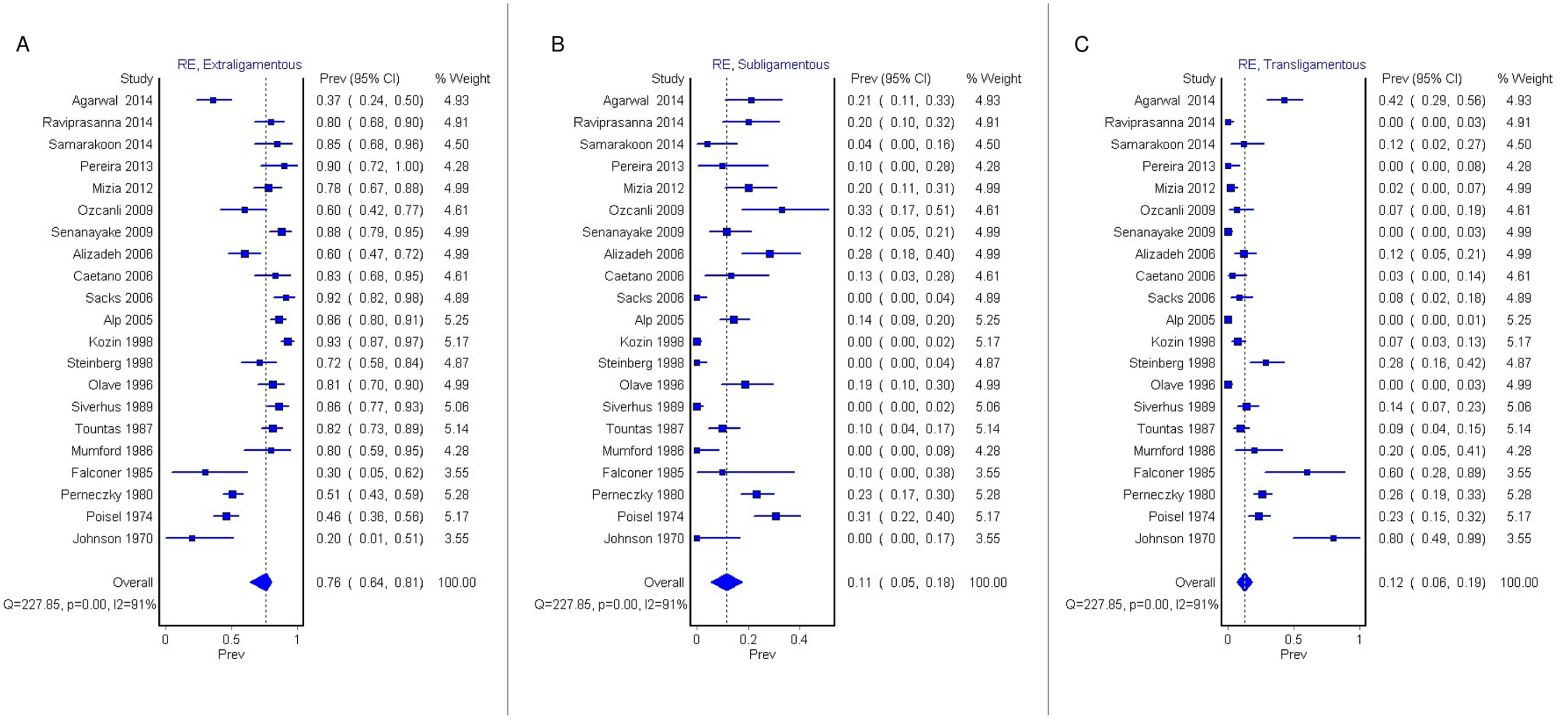
Forest plots for pooled prevalence of extraligamentous (A), subligamentous (B), and transligamentous (C) types of TMB in the subgroup of cadaveric studies. RE—Random Effects.

**Fig 5:**
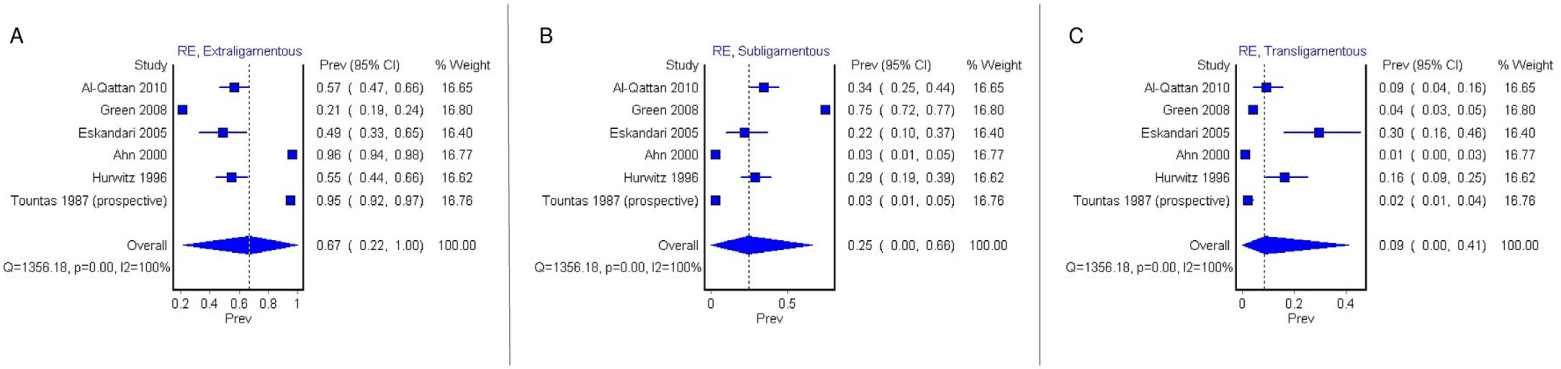
Forest plots for pooled prevalence of extraligamentous (A), subligamentous (B), and transligamentous (C) types of TMB in the subgroup of intraoperative studies. RE—Random Effects.

The pooled prevalence of Lanz group 1 did not differ significantly based on the type of study. In the subgroup analysis of the 21 cadaveric studies only (n = 1250 hands) [2, 3, 8, 9, 16, 17, 19, 21, 23, 24, 26–29, 31–37], the pooled prevalence of extraligamenous (Fig 4A), subligamentous (Fig 4B), and transligamentous (Fig 4C) types were 76.4% (95% CI: 63.9%- 81.1%), 11.4% (95% CI: 5.5%- 7.7%), and 12.2% (95% CI: 6.1%- 18.6%), respectively (Cochran's Q, p = 0.000; I^2^ = 91.2%, 95% CI: 88.0%- 93.6%). In the subgroup analysis of the 6 intraoperative studies (n = 2256) [6, 7, 10, 20, 22, 37], the pooled prevalence of extraligamenous (Fig 5A), subligamentous (Fig 5B), and transligamentous (Fig 5C) types were 66.8% (95% CI: 21.0%- 100%), 24.5% (95% CI: 0%- 65.8%), and 8.7% (95% CI: 0%- 40.9%), respectively (Cochran's Q, p = 0.000; I^2^ = 99.6%; 95% CI: 99.5%- 99.7%).

Further subgroup analysis was performed based on the geographical region of the included studies. In 8 studies (n = 2039 hands) from the United States of America [10, 21, 23, 24, 26, 32, 35, 37] the pooled prevalence of the extraligamentous type was 73.5% (95% CI: 29.9%- 100%). The second most common course was the transligamentous type with a pooled prevalence of 19.4% (95% CI: 0%- 55.0%), which was higher than that of the subligamentous type which had a pooled prevalence of 7.1% (95% CI: 0%- 34.5%; Cochran's Q, p = 0.000; I^2^ = 99.4%, 95% CI: 99.3%- 99.5%), although the difference was not statistically significant.

In the subgroup of 7 European studies (n = 611 hands) [3, 8, 9, 17, 20, 22, 28] the pooled prevalence of extraligamentous, subligamentous, and transligamentous courses were 63.7% (95% CI: 44.3%- 77.8%), 24.6% (95% CI: 10.5%- 40.2%), and 11.7% (95% CI: 2.3%- 24.9%), respectively (Cochran's Q, p = 0.000; I^2^ = 94.3%, 95% CI: 90.5%- 96.5%). While in the subgroup of 8 Asian studies (n = 747 hands) [2, 6, 7, 16, 31, 33, 34, 36], the pooled prevalence of extraligamentous, subligamentous, and transligamentous courses were 78.7% (95% CI: 54.7%- 90.1%), 12.6% (95% CI: 1.5%- 28.5%), and 8.7% (95% CI: 0.1%- 23.4%), respectively (Cochran's Q, p = 0.000; I^2^ = 96.1%, 95% CI: 94.0%- 97.4%).

To explore the role of sample size in the high heterogeneity among the included studies, a sensitivity analysis was performed on 8 studies (n = 2644 hands) of which each had a sample size of ≥ 100 hands [3, 6, 7, 9, 10, 17, 24, 37]. Sensitivity analysis found the pooled prevalence of extraligamentous, subligamentous, and transligamentous courses of the TMB to be 74.4% (95% CI: 38.2%- 98.4%), 18.6% (95% CI: 0%- 48.7%) and 7.0% (95% CI 0%- 29.6%), respectively (Cochran's Q, p = 0.000; I^2^ = 99.6%, 95% CI: 99.5%- 99.6%).

### Bilateral Hand Symmetry in Course of the TMB

Analysis of 4 intraoperative studies [7, 10, 22, 35] consisting of a total of 423 patients undergoing bilateral carpal tunnel release surgery, found that 72.3% (95% CI: 58.4%- 84.4%) of patients had the same course of the TMB bilaterally, while 27.7% (95% CI: 15.6%- 41.6%) of patients had different courses of the TMB on each side (Cochran's Q, p = 0.000; I^2^ = 85.0%, 95% CI: 62.8%- 94.0%).

### Side of Branching of the TMB

A total of 13 studies (n = 1394 hands) reported data on the side of branching of the TMB (Table 2) [7–9, 16, 19, 20, 22, 24–26, 30, 32, 37]. The pooled prevalence for ulnar side of branching was 2.1% (95% CI: 0.9%- 3.6%), while the pooled prevalence for radial/anteroradial side of branching was 97.9% (95% CI: 96.4%- 99.1%; Cochran's Q, p = 0.02; I^2^ = 61.0%, 95% CI: 30.0%- 78.2%).

**Table 2:** Pooled results for ulnar side of branching, Lanz group 2, 3, and 4.

Study	Type	n =	Ulnar side of branching n = (%)	Lanz group 2 n = (%)	Lanz group 3 n = (%)	Lanz group 4 n = (%)
**Agarwal 2014 [2]**	C	52		2 (3.8%)	6 (11.5%)	1 (1.9%)
**Planitzer 2013 [30]**	C	21	3 (14.3%)			
**Mizia 2012 [8]**	C	60	2 (3.3%)			
**Alizadeh 2006 [16]**	C	60	7 (11.7%)	11 (18.3%)		11 (18.3%)
**Caetano 2006 [19]**	C	30	3 (10.0%)			
**Sacks 2006 [32]**	C	48	0 (0.0%)			
**Barbe 2005 [18]**	C	75		0 (0.0%)	2 (2.7%)	0 (0.0%)
**Eskandari 2005 [20]**	IO	37	0 (0.0%)			
**Ahn 2000 [7]**	IO	354	4 (1.1%)		1 (0.3%)	
**Kozin 1998 [24]**	C	101	0 (0.0%)			
**Hurwitz 1996 [22]**	IO	74	1 (1.4%)			
**Olave 1996 [27]**	C	60			1 (1.7%)	
**Mackinnon1988 [25]**	C	50	0 (0.0%)		1 (2.0%)	
**Tountas 1987 [37]**	IO	286	5 (1.7%)	2 (0.7%)	6 (2.1%)	6 (2.1%)
**Tountas 1987 [37]**	C	92	0 (0.0%)	2 (2.2%)		0 (0.0%)
**Falconer 1984 [21]**	C	10		2 (20.0%)		0 (0.0%)
**Mumford 1986 [26]**	C	20	0 (0.0%)			
**Perneczky 1980 [9]**	C	163	3 (1.8%)	8 (4.9%)		
**Lanz 1977 [1]**	IO	246		18 (7.3%)	7 (2.8%)	4 (1.6%)
**Pooled prevalence**			2.1%(95%CI:0.9%- 3.6%)	4.6%(95%CI: 1.6%- 9.1%)	2.6%(95%CI: 0.1%- 2.8%)	2.3%(95%CI: 0.3%- 5.6%)

C- cadaveric, IO—intraoperative.

In the subgroup analysis of 4 intraoperative studies (n = 749 hands) [7, 20, 22, 37], the pooled prevalence of ulnar side of branching of the TMB was 1.5% (95% CI: 0.7%- 2.5%; Cochran's Q, p = 0.857; I^2^ = 0%, 95% CI: 0%- 40.1%), while in the subgroup of 10 cadaveric studies (n = 645) the pooled prevalence of ulnar side of branching was 2.6% (95% CI: 0.6%- 5.6%; Cochran's Q, p = 0.000; I^2^ = 71.6%, 95% CI: 46.0%- 85.1%).

### Lanz Group 2—Accessory Branches of the Median Nerve Distal to the Carpal Tunnel

Seven studies (n = 984 patients) [1, 2, 9, 16, 18, 21, 37] counted the frequency of accessory branches of the median nerve distal to the carpal tunnel (Table 2). The pooled prevalence of this variation was 4.6% (95% CI: 1.6%- 9.1%; Cochran's Q, p = 0.000; I^2^ = 84.4%, 95% CI: 71.0%- 91.6%). In the subgroup analysis of 6 cadaveric studies (n = 452 hands) [2, 9, 16, 18, 21, 37] the pooled prevalence was 5.3% (95% CI: 1.2%- 11.6%; Cochran's Q, p = 0.000; I^2^ = 80.1%, 95% CI: 56.7%- 90.8%), while in subgroup analysis of 2 intraoperative studies (n = 532 hands) [1, 37], the pooled prevalence was 3.4% (95% CI: 0%- 11.5%; Cochran's Q, p = 0.000; I^2^ = 94.4%, 95% CI: 82.7%- 98.2%).

### Lanz Group 3—High Division of the Median Nerve

Seven studies (n = 1123 hands) [1, 2, 7, 18, 25, 27, 37], measured the prevalence of a high division of the median nerve which was found to be a rare variation, with a pooled prevalence of 2.6% (95% CI: 0.1%- 2.8%; Cochran's Q, p = 0.003; I^2^ = 70.3%, 95% CI: 35.0%- 86.4%) (Table 2). Five of the 7 studies also reported data on the rate of a PMA in the presence of a high division of the median nerve [1, 2, 7, 18, 37]. Of the 22 hands reported in the five studies with Lanz group 3 variation, the prevalence of a PMA was 63.0% (95% CI: 42.6%- 81.4%; Cochran's Q, p = 0.473; I^2^ = 0%, 95% CI: 0%- 76.5%).

In subgroup analysis of the 4 cadaveric studies, (n = 237 hands) [2, 18, 25, 27], the pooled prevalence of Lanz group 3 was 4.1% (95% CI: 1.2%- 8.6%; Cochran's Q, p = 0.118; I^2^ = 48.9%, 95% CI: 0%- 83.1%), while in the subgroup of 3 intraoperative studies (n = 886 hands) [1, 7, 37], the pooled prevalence was 1.4% (95% CI: 0.2%- 3.6%; Cochran's Q, p = 0.014; I^2^ = 76.4%, 95% CI: 22.8%- 92.8%).

### Lanz Group 4—Accessory Branches of the Median Nerve Proximal to the Carpal Tunnel

Six studies (n = 821 hands) counted the frequency of accessory branches of the median nerve proximal to the carpal tunnel (Table 2) [1, 2, 16, 18, 21, 37]. The pooled prevalence of this variation was 2.3% (95% CI: 0.3%- 5.6%; Cochran's Q, p = 0.000; I^2^ = 78.6%, 95% CI: 55.9%- 89.6%). In subgroup analysis of 5 cadaveric studies (n = 289 hands) [2, 16, 18, 21, 37], the pooled prevalence of Lanz group 4 was 3.1% (95% CI: 0%- 9.8%; Cochran's Q, p = 0.000; I^2^ = 85.6%, 95% CI: 68.2%- 93.5%), while in subgroup analysis of 2 intraoperative studies (n = 532 hands) [1, 37], the pooled prevalence was 2.0% (95% CI: 0.9%- 3.3%; Cochran's Q, p = 0.716; I^2^ = 0%, 95% CI: 0%- 0%).

### Hypertrophic Thenar Muscles and Its Relationship to TMB

Four studies (n = 1934 hands) [6, 7, 10, 22] examined the frequency of a hypertrophic thenar muscle running over TCL (Table 3). The pooled prevalence of this variation was 18.2% (95% CI: 6.8%- 33.0%; Cochran's Q, p = 0.000; I^2^ = 96.8%, 95% CI: 94.3%- 98.2%). In the 458 hands with the presence of hypertrophic thenar muscles in these studies, the pooled prevalence of extraligamentous, subligameous, and transligamentous courses of the TMB were 64.7% (95% CI: 36.3%- 80.9%), 11.8% (95% CI: 0.3%- 29.8%), and 23.4% (95% CI: 5.4%- 43.4%), respectively (Cochran's Q, p = 0.000; I^2^ = 88.0%, 95% CI: 71.6%- 94.9%). In comparison, in the 1475 hands without a hypertrophic thenar muscles in these studies, the pooled prevalence of extraligamentous, subligamentous, and transligamentous TMB courses was 50.2% (95% CI: 0%- 100%), 48.2% (95% CI: 0%- 100%), and 1.7% (95% CI: 0%- 100%), respectively (Cochran's Q, p = 0.000; I^2^ = 99.9%, 95% CI: 99.8%- 99.9%).

**Table 3:** Prevalence of hypertrophic thenar muscles and its associated course of the thenar motor branch.

Study	n = (% HT)	HT thenar muscle			Non-HT thenar muscle		
		ELn = (%)	SLn = (%)	TLn = (%)	ELn = (%)	SLn = (%)	TLn = (%)
Al-Qattan 2010 [6]	100 (40.0)	36(80.0)	0 (0.0)	9(20.0)	30(46.9)	34(53.1)	0(0.0)
Green 2008 [10]	1400 (27.6)	299(77.5)	29(7.5)	58(15.0)	0(0.0)	1014(100.0)	0(0.0)
Ahn 2000 [7]	354 (8.5)	20(66.7)	7(23.3)	3(10.0)	320(98.8)	3(0.9)	1(0.3)
Hurwitz 1996 [22]	80 (8.8)	0(0.0)	3(42.9)	4(57.1)	44(60.3)	20(27.4)	9(12.3)
Total n = (PP (%))	1934(18.2)	355(64.7)	39(11.8)	74(23.4)	394(50.2)	1071(48.2)	10(1.7)

HT—hypertrophic; EL—Extraligamentous TMB; SL—Subligamentous TMB; TL—Transligamentous TMB; PP = pooled prevalence.

## Discussion

Despite the high prevalence of variations of the median nerve in the carpal tunnel reported in the literature, few major anatomical textbooks make reference to such variations [2]. Of particular surgical importance, is the highly variable course of the TMB, that when damaged, can leave patients with functional deficits of the thumb including thenar weakness and loss of opposition [38]. Our meta-analysis found that the most common course of the TMB was extraligamentous with a pooled prevalence of 75.2%. This was true in all but 3 [10, 21, 23] of the 27 included studies. Of particular noteworthiness, were the results from an intraoperative study by Green and Morgan [10], who found 74.5% of 1400 hands to be of the subligamentous course. Their results significantly differ from the rest of the included studies, and we suspect that this could be due to the intraoperative nature of the study, in which the limited scope of the visual field during surgical procedures may hinder the ability of the investigator to accurately identify the course of the TMB.

It has been suggested by Lanz and others that a transligamentous course of the TMB may be associated with an increased risk of CTS [1, 9]. While not all of the included intraoperative studies looked exclusively at people undergoing CTR, it was interesting to find that the prevalence rate of the transligamentous course was slightly higher in the cadaveric subgroup than in the intraoperative subgroup (10.3% vs. 8.7%). Furthermore, the prevalence of a subligamentous course of the TMB was more common in the subgroup of patients undergoing intraoperative procedures than in the subgroup of cadaveric studies (24.59% vs. 13.9%). We suspect that a subligamentous course of the TMB may increase compression of the median nerve, similar in mechanism to the reported increased compression caused by a bifid median nerve, which has been reported to increase the risk for development of CTS [39, 40].

The prevalence of a subligamentous course of the TMB was found to be higher in Europeans (24.6%) than in Asians (12.6%) and Americans (7.1%). The prevalence of a transligamentous pattern however, was higher in Americans (19.4%) than in Europeans (11.7%) and Asians (8.7%). It should be noted though, that due to significant heterogeneity, no statistically significant differences were found between the subgroups based on geographical origin of the studies.

The prevalence of a transligamentous course of the TMB ranged from 0% [17, 27, 29, 31] to 80% [23] in the included studies. It has been hypothesized that the high variance of this course may be due to race or due to the association of the transligamentous course with CTS and intraoperative studies [6]. However, we found no such associations in our analysis. Kozin et al. [24] explained that thin oblique fibers should not be considered as the TCL, and that an extraligamentous TMB piercing such fibers may have been reported as transligamentous by some authors. While this may be in part true, we believe that the highly variable nature of TMB itself, may account for these differences.

There are few, if any, clinical or electrophysiological signs that anatomical variations of the median nerve in the carpal tunnel are present in a patient [41]. While physical examination using the middle finger flexion test may be accurate for predicting the location of the median nerve in the presence of an extraligamentous course, it is not able to accurately do so in the case of a transligamentous course, and thus provides no way of determining what course variant of TMB a patient may have [38]. We found the pooled prevalence of the transligamentous course of the TMB to be 11.3%. This course is at the highest risk for inadvertent damage during CTR surgery [42]. As nearly one in ten patients undergoing CTR surgery may have a transligamentous course of the TMB, we support the 1977 recommendation by Lanz [1] for the use of only an ulnar surgical approach, with a careful layer by layer dissection of the carpal tunnel to avoid neurovascular injury. A subligamentous course of the TMB is also at increased risk of division during surgical procedures, and care should be taken to identify the presence of this variation [41]. Additionally, while rare, an ulnar side of branching of the TMB does exist in 2.1% of the population, and is at increased risk of injury during CTR procedures [7–9]. Furthermore, the incidence of bilateral CTS is high, with 59% [43] to 87% [44] of patients presenting with bilateral symptoms. When performing a bilateral CTR, surgeons should keep in mind that symmetry in the course of TMB between hands occurs only in 72.3% of individuals.

Additionally, the presence of hypertrophic muscle over or interposed within the TCL, prevalent in 18.2% of hands, should serve as a warning sign for potential TMB variants. The pooled prevalence of the transligamentous course was 23.4% in patients with hypertrophic muscle over the TCL, as compared to 1.7% without it. The source of the muscle tissue over or within the TCL is not known [6]. Some have hypothesized that it is a broad origin of the flexor pollicis brevis and the flexor digiti minimi from the TCL or an ulnar extension of thenar muscles to the TCL [23], while others have believed it to be an aberrant muscle [10]. Regardless of the origin, our findings support the correlation originally postulated by Mannerfelt and Hybbinette [11], that the presence of hypertrophic muscles is associated with TMB variants, and as such surgeons should take caution when encountering it.

**Fig 6:**
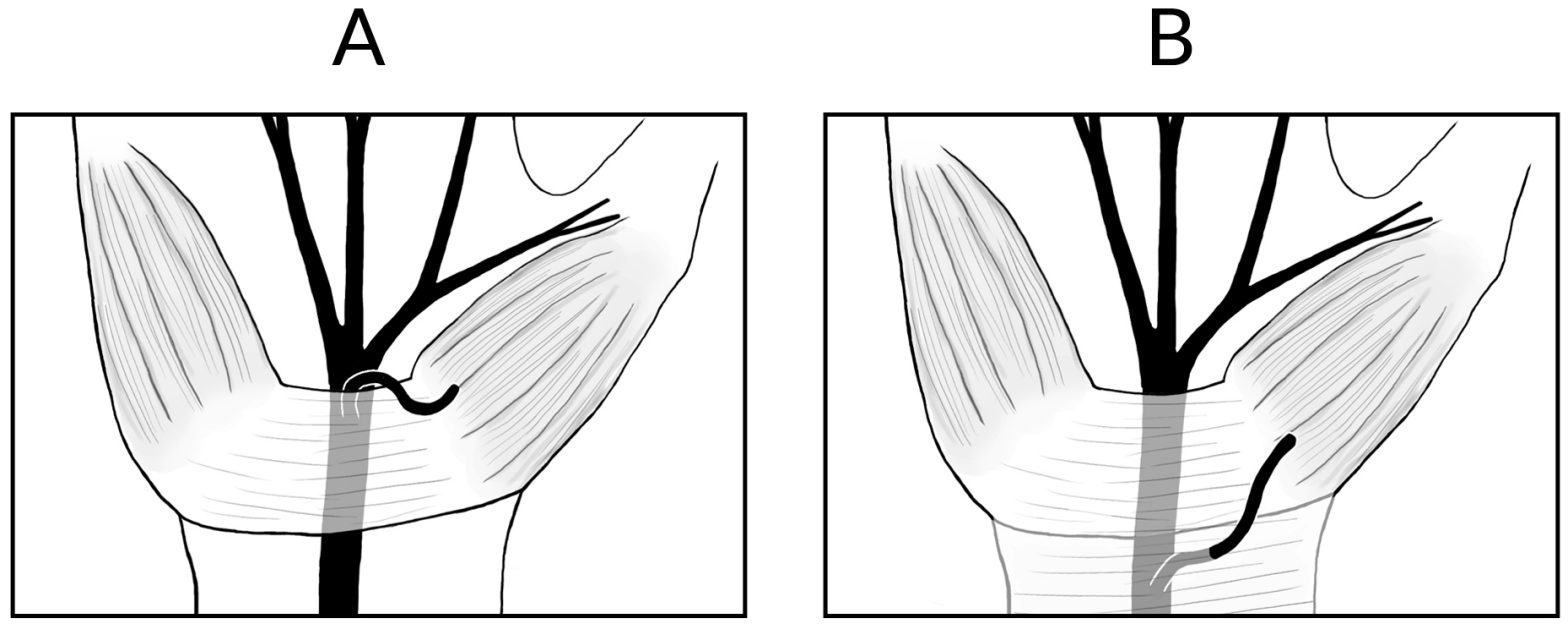
Supraligamentous course of the TMB (A), preligamentous course of the TMB (B).

Besides extraligamentous, subligamentous, and transligamentous, other more rare anomalies in the course of the TMB have been described [1, 6]. Lanz described the finding of a supraligamentous course of the TMB (Fig 6A), which consists of the TMB running over the top of the TCL ligament to reach the thenar muscles [1, 14]. Additionally, a preligamentous course of the TMB (Fig 6B) has been reported in several studies [6, 7, 17, 27, 31]. In this course, the TMB arises proximal to the TCL and runs a superficial course over the TCL to reach the thenar muscles [6]. Both preligamentous and supraligametous courses would be at an increased risk of damage in the event of traumatic injury to the wrist or during CTR, further emphasizing the need for careful visualization of the TMB during surgery.

Lanz additionally described accessory branches of the median nerve arising both proximally (Lanz group 4) and distally (Lanz group 2) to the carpal tunnel [1]. We found the pooled prevalence of both of these variations to be low, with the prevalence of Lanz group 2 and group 4 at 4.6% and 2.3%, respectively. While these accessory branches are primarily sensory in function, care should be taken to avoid iatrogenic injury to the nerves due to the risk of sensory loss and neuromata development [1].

The prevalence of Lanz group 3, a high division of the median nerve resulting in a bifid median nerve, was found to have a pooled prevalence of 2.6%. Lanz reported the two parts of this variant to have nearly equal diameter [1]. However, other reports in the literature have found varying sizes with both predominant radial and ulnar parts [45, 46]. A bifid median nerve has been reported to be often associated with aberrant nerve branches, and as such, requires careful dissection to avoid injury to these branches [45].

A bifid median nerve, likely due to increased cross sectional area as compared to a normal median nerve, has been found to be associated with an increased rate of CTS [39, 40]. In an ultrasound study by Bayrak et al. [39], the prevalence of a bifid median nerve was 19% in patients with CTS as compared to only 9% of healthy controls. However, conflicting ultrasound studies have found no significant difference in the prevalence of a bifid median nerve between healthy controls and patients with CTS [47]. Interestingly, in the current analysis the prevalence of a bifid median nerve was lower in the intraoperative subgroup (1.4%) than in the cadaveric subgroup (4.1%). Thus, while some ultrasound studies have supported the association of a bifid median nerve and an increased risk of CTS, these findings are not supported by evidence from intraoperative studies. It may be suspected that this difference may be partially due to the limited visual scope of the surgical field during the procedures. However, the pooled prevalence rate of a bifid median nerve in our cadaveric subgroup, is still far lower than the 9% [39] to 15.4% [47] reported in ultrasound studies for healthy controls.

Lanz reported that the presence of a bifid median nerve was often associated with the presence of a PMA running between the two parts of the bifid nerve [1]. In our meta-analysis, we found that Lanz group 3 was associated with a PMA in 63.0% of hands. The median artery in utero is the primary source of blood supply to the hand, but it regresses throughout embryonic development [45]. A PMA, with a prevalence ranging from 10% to 50% depending on the population, when present supplies blood to the superficial palmar arch or to the radial digits in addition to the median nerve [45]. The presence of PMA has been associated with CTS and damage to this artery during CTR may significantly affect circulation in the forearm and hand [2]. Moreover, PMA has been found to be highly associated with anatomical variants of the median nerve in both the forearm and hand [2]. As both a bifid median nerve and a PMA are common anatomical variants that are often associated with other median nerve variants, ultrasound screening of patients before undergoing CTR surgery can help identify those at increased risk of iatrogenic nerve damage and allow for better surgical planning [48].

Our meta-analysis was limited by the high heterogeneity between studies, the poor quality of some of the included studies, the lack of a proper quality assessment for anatomical meta-analysis and slightly different definitions of anatomical variations and interpretations of both Poisel's classification [3] and Lanz's classification [1] that was used in individual studies. Furthermore, due to the lack of a proper measure for multi-categorical prevalence, no publication bias assessment was performed. To limit bias in our analysis, we contacted authors when possible to explain all inconsistencies arising from the data of the included studies.

Despite subgroup analysis by both study type and geographical distribution, as well as a sensitivity analysis that included studies with ≥ 100 hands, no source of the high heterogeneity could be identified. However, high heterogeneity was not consistent throughout our study, with the I^2^ ranging from as high as 99.9%, to as low as 0%. We suspect that some heterogeneity could be due to the different anatomical definitions used in the individual studies, and, as such, to limit heterogeneity among future studies, we strongly recommend the use of the same anatomical definitions of variations as used in this analysis. Moreover, as it has not been explored in previous studies, the role of gender as a cause of heterogeneity cannot be ruled out. As such future studies should explore the association between gender and median nerve variations. However, we suspect that the principal cause of heterogeneity is due to the highly variable nature of the course of TMB and the median nerve in the carpal tunnel. As such, we recommend that surgeons proceed with careful consideration and attention to the median nerve and its TMB while operating in the region of the carpal tunnel.

## Conclusions

Anatomical variations of the median nerve in the carpal tunnel and the course of its TMB are prevalent among the population. The course of the TMB is highly variable, and while the most common course is the extraligamentous type, variants at high risk of iatrogenic damage during CTR or repair of traumatic injuries often occur. Hypertrophic thenar musculature over the TCL is associated with a higher rate of TMB variants and should serve as a warning sign for surgeons. Preoperatively, the use of ultrasound can help to identify patients with a bifid median nerve or PMA, who are more likely to have median nerve variations. Our findings support the use of an ulnar side approach to CRL to prevent inadvertent damage to the median nerve and its branches.

## Supporting Information

S1 File PRISMA 2009 Checklist. (PDF)

S1 Table Complete Data Set. (XLSX)
