# Correction: The Prevalence of Anatomical Variations of the Median Nerve in the Carpal Tunnel: A Systematic Review and Meta-Analysis

**DOI:** 10.1371/journal.pone.0138300

**Published:** 2015-09-11

**Authors:** The PLOS ONE Journal Staff

The Funding section appears incorrectly. Please view the complete, correct Funding statement here: The publication of this manuscript has been funded by the Jagiellonian University Medical College, Faculty of Medicine KNOW (Leading National Research Center) funds.

The publisher apologizes for the error.
